# Pennsylvania State Dental Society

**Published:** 1889-09

**Authors:** 


### Reports of Society Meetings.

### PENNSYLVANIA STATE DENTAL SOCIETY.

*Tuesday, July 30, 1889.—Afternoon Session.*

TWENTY-FIRST annual meeting, held at Cresson, Pa., July 30, 1889.

### ADDRESS OF THE PRESIDENT, DR. H. C. REGISTER, PHILADELPHIA, PA.

Another period marks the history of time since this association joined in mutual well-doing; and amidst the vicissitudes that have ebbed and flowed for the weal or woe of mankind, a deluge has wasted one of the most beautiful valleys far up in the mountains of our State, not far from this place of meeting. Thousands of people have been swept into eternity under the most appalling circumstances, while to-day the Conemaugh flows peacefully on by the mountain-side with its ripples and shadows in beautiful play. A multitude of people mourn for those who are not, and with commendable spirit have commenced the rebuilding of waste places and broken fortunes.

A calamity like that of Conemaugh touches the vital sympathy of a nation,—comes home to every man as though the sufferer were his neighbor, and his hand goes to his pocket, and from his abundance or out of his poverty he is willing to help as best he may.

What a grand sight to see a nation thus giving, unexampled in the world for generosity, promptitude, and systematic wisdom of distribution, showing as nothing else could the intelligence, quickness, and courage in the permanent strength of the American character! While we are proud of the citizens who have shown themselves so worthy to meet so great a disaster, we should remember specially those who are of our own fraternity. In sore distress and complete loss of strength the helping hand becomes necessary until convalescence is passed. So it is with Johnstown to-day. A city of twenty thousand population has been broken to its foundation. A happy and prosperous community no longer exists, and among its sufferers were ten dentists: one lost his life, and left a destitute family thrown upon the world, the others lost members of their families, and all lost their property. There has been a circular card issued to the profession asking help for these stranded members of the dental profession and their families. It is a noble opportunity, and I trust the members of this association, one and all, will appreciate the call as a personal solicitation!

To this association, composed as it is of gentlemen representing every part of the State,—the county, village, town, and city,—I deem it a fitting opportunity to speak to you briefly of the latest theory in its application to decay of the teeth, for the reason that after much pruning it continues a vigorous growth and is bearing fruit in the laboratory, the clinic-room, and yielding a rich harvest in the field of practice.

To-day we have presented to the profession one of the most interesting theories ever launched upon the sea of medicine,—viz., the germ theory, or, as some writers put it, *Bacteriopathy.* Micro-organisms fill a large territory; they are vegetable parasites, and claim our special attention as dentists.

Cullen says, "There are more false facts than false theories in medicine;" but if we review its history, or that of dentistry, we find much that is false both in theory and in fact. While dentistry is ever progressing, reaching out to grasp the true means leading to better and surer results, the veil of revelation lifts slowly, and we continue to swing backward and forward with pendulum-like regularity in the slow movement upward. Thompson told us, many years ago, disease was the product of cold, and heat was the panacea to all ills. Thompsonian ideas to-day are *nil,* but its legacy to medicine continues to flavor many prescriptions.

History is ever repeating itself; the same sun rises and sets, the same humanity is ever looking for happiness. In each revolution of time, science makes an impression that finds unchangeable conditions which give us an intimation of the indelible language of Caro.

Astrology, that claimed the greatest minds of earlier ages that the future might be held within the grasp of man, led up to the splendid results of astronomy!

Alchemy, in its control of mighty philosophers, in that the king of metals, gold, might be created from the baser kind, and the elixir of youth be perpetuated forever, brought forth the splendid achievements of chemistry.

And so, through all experience of time, man is striving upward and onward, not in the present as in the olden time to create facts, but to reveal them. The skeins of life are woven in the universe; we cannot change conditions or things; revelation is for man, and he stands as the most sublime problem of it all, and to know nature is to interpret one's self.

The most universal disease the dentist has to combat is tooth-decay. This has been accepted as an external attack upon the tooth, and progressing inward until the organ is lost. To intelligently appreciate the conditions leading up to this universal disease and the means of staying its progress, it is essential to familiarize one's self with the tissues we treat, and to have an understanding of the environment or atmosphere in which the organ resides. A knowledge of the active or passive nature of the parts is necessary, and how the environment is created and the influences leading up to and controlling it and its after-influence upon surrounding tissues. All organic conditions begin as simple forms. In life we have the simple segment or cell as the genesis to all animal creation. Take, for instance, the seed: how little do we know, after all investigation and knowledge, of the formative power residing in it. The seed is primitive in comparison with its relations to plant-life; yet what mystery shrouds its creation in continuance of its kind, and still higher and grander in its essential relationship in the problem of all animal life. Metaphysics has not reached it; man must first unravel the affinities of matter and view the structure as it stands before him, one grand, sublime whole.

The teeth we find to commence as simple homogeneous tissue, and to pass into complex heterogeneous tissue. While they are but a small part of the human economy within themselves, the relationship is so intimate and commingled that we cannot appreciate the one without following the current of study into the general circulation of formation, growth, and maintenance of life in general.

The permanent set of teeth are the only organs formed and perfected after birth; they develop from the simple papilla of a jelly-like consistency into the hardest effort of nature. During the development of these organs, the life-forces hold them in hand and seemingly are able to create proper building-material out of any life-sustaining nutrients. From the papillæ we have the pulp. Around its surface are developed a layer of cells called the odontoblasts, yet being most intimately a part of the pulp. From this odontoblastic layer we have the growth of the dentine, being deposited in successive layers, as though a growth was made and a time of rest taken; this is shown by the incremental lines, and can be tested by soaking a tooth in caustic potash, when the dentine can be separated in concentric rings. In these spaces the matrix or basis-substance is not so perfectly calcified and particularly so at its termination, where it joins the enamel prisms. The cap or dome fitting the conformations of a tooth-crown have many interglobular spaces, which continue after the tooth is erupted. Throughout the dentine are holes,—you know them as the tubuli; they are very small indeed, requiring about two hundred placed alongside of each other to be as thick as the hair of a man's beard. In the tubuli we have the fibrillæ and the pain experienced in cutting dentine, that you have been in the habit of calling sensitive dentine, which sensation is produced, as I believe, by the bruising of minute nerve-fibres.

Thus far do we see a tooth is made up of a very complex foundation; the fibrillæ are really prolongations of the odontoblasts, and are distributed throughout the tooth in most profuse richness, giving to the organ that delicate tactile sensibility that exceeds the same sensation in the finger-tips.

In young teeth the fibres continue into the finest ramifications, and pass through the protoplasmic material found in the interglobular spaces, which later in life are terminated by calcification. The termination of the tubuli is mainly in anastomosing with the lateral branches, but many extend into the lacunæ and canaliculi of the cementum. It is, to my mind, the ignoring of this anatomical condition that causes so many unsuccessful devitalized cases of long standing.

In this condition of health we find the teeth externally subjected to retrogression through fermentation.

A ferment is regarded as being composed of organized living beings, either of an animal or vegetable origin.

In studying the organization of the most minute living things, naturalists have at all times been very much puzzled to know if they had to deal with animal or plant life. If we see a horse, a tree, or a shrub, they are readily classified, each to its own order, but with organisms studied under the lens it is not so easy; and it was this uncertainty of things that brought forth the word *microbe* by the eminent French surgeon, Sedillot, about eleven years ago. Up to this time naturalists—even such a high authority as Heckel—accepted an intermediate kingdom of living things, and called it Protista. It formed the connecting-link in evolution.

The word microbe simply means small living things, but decides nothing as to their being animal or vegetable. Pasteur has accepted the word in his writings. Microbes play a most important part in nature, their chief office being apparently to reduce complex constituents into simple substances, they being nourished at the expense of organic matter. The study of microbes concerns all mankind; for while many of the effects of these have not been clearly proven, our best authorities are now combining cause and effect so closely that the health of a nation or a continent has been shown largely to hinge upon the proper understanding of these low forms.

In the mouth of animals we have a natural culture-apparatus for a certain variety of micro-organisms. Many of these are not pathogenic, and have no deleterious influence upon the general health, while other varieties are specially designated by the disease they produce. Miller and Black have most satisfactorily shown the action of bacteria upon the teeth by producing caries artificially, and it has also been shown by Davaine that anthrax or splenic fever is directly due to microbes.

Medicine will furnish an easy solution to many problems, when theories become proven facts, and the veil of empiricism is lifted and the medical profession placed upon the very pinnacle of the most humane of sciences.

The reason why animals generally do not lose their teeth is from the absence of a ferment; this is the secret of the whole origin of tooth-decay. Acids may denude the surfaces by decalcification, but not produce decay. Quadrupeds are, in their natural state, free from conditions to form a ferment in the mouth, but man is not so fortunate, and the higher his plane of civilization the more general become the spores of minute organisms, for the simple reason that what comports with his idea of comfort may give origin to these destroying growths. In illustration, I need only refer to the so-called conveniences in drainage and the general methods of heating.

Decay of the teeth, as I have stated, comes from a ferment upon the outside which attacks the lime-salts through affinity; and in these little places upon the teeth the parasitic germs are implanted, take root, as it were, and grow, existing by and through the decomposition of the tooth-substance. They force their way into any broken-down space and growth, producing lactic acid, which takes hold of the lime-salts, and thus a constant increase of plant-growth is possible; and so it proceeds, with but a passive resistance from nature, until the pulp is exposed.

Under these conditions we see at once how necessary it is to make a perfectly tight filling to check the decay, and also how, through thermal changes, sensation is carried by metal fillings to the odontoblastic ganglion and the sensorium. These conditions may be ameliorated by the insolation of all metal fillings by a gutta-percha solution and a careful sanitation of the mouth by antiseptics.

The atomization of the mouth and teeth daily in connection with brush and stick, constitute the preventive means. A word to the wise is sufficient, for to know the etiology of a disease enables us to meet and treat it successfully.

### DISCUSSION ON DR. JOSEPH HEAD'S PAPER ON "DENTAL ASSERTION AND DISCUSSION."

*Dr. E. C. Kirk*, Philadelphia.—I have nothing to say regarding the paper except to endorse it. I think a great deal is brought before dental conventions that is open to criticism, as Dr. Head states. Some individuals do not know the truth, while others simply do not have the ability to tell it. Some of the statements made are often a hinderance to our progress. I think the doctor's argument holds water thoroughly, even better than his favorite cosmoline canal-stopping.

*Dr. W. E. Magill*, Erie.—It strikes me that what we ought to think of is the apparent necessity for such a paper. If we are in our discussions as we are in our practice, open to such criticism, it is well enough for us to inquire where the trouble originates, who is responsible for it, and whether there is any proper cure. From what experience I have had and observation I have made, it lingers upon us as part of the historic past. There was a time in dentistry when there was no beaten track, when every man who investigated did so as an originator,—originator of the principles. There was no man before him who had taken any steps in advance, so you will readily understand how men in the earlier profession, fully occupied in their professional duties, together with the increased sphere of usefulness of the profession, were situated. He had his hands full, and in looking into the subject I think we may ask for charitable criticism. The profession of to-day is different, and it is a question whether they are in need of the same charity; with its enlarged advantages and free investigations, our men should have the time and right to observe, and, I think, should be held to a closer degree of responsibility for their attainments.

It is now very different from what it was twenty-five or thirty years ago, and I think if we fail to recognize the advance of the profession and increase among us, we shall fail to perform our duty. I think Dr. Head has done us a good in mentioning one of our faults that most of us are guilty of,—awake to our virtues, but not awake to our deficiencies.

*Dr. C. V. Kratzer.*—There is one paragraph in Dr. Head's paper I can hardly endorse. I think he made a charge against the profession he cannot substantiate; when he charges men of the society with self-importance and self-righteousness in their modes of practice I do not think he does what is entirely proper, or what could be borne out upon investigation. In the society meetings which I attend it has not been my experience that men have advanced their theories and insisted that they are the correct ones. I have noticed a generosity of sentiment that the doctor has not accorded the profession. In our societies there is a change of opinions and theories, and men are given credit by other members for ability and judgment, as they give themselves credit. He mentioned a certain meeting where members got up and advanced theories of filling canals, one with cotton, another with oxyphosphate, and another with gutta-percha, and that each one insisted his was the only proper method of procedure. Now, while this may have been the case in that particular meeting, it is not so in those I have attended. Then he says that members should demonstrate their methods by actual experiments, and that is the only way to establish the correctness of their methods. It is all very well, but I think it is a difficult matter to bring before dentists these practical illustrations. While it can be done to back molar teeth out of the mouth and imbedded in plaster, when the length of the roots can easily be measured, and the extent of the foramen ascertained, it is much more difficult in the mouth. We have that plaster tooth before us and we can turn it into any position we desire, and we can have direct access into the roots. It is far more difficult to do this in the mouth, and still more difficult to bring work done in the mouth before the society.

*Dr. Joseph Head*, Philadelphia.—My reason for suggesting experiments on teeth in plaster is, it would be well known that if dentists failed to perform their operations on teeth in plaster they could not perform more difficult operations in the mouth.

*Dr. L. A. Faught*, Philadelphia.—I think Dr. Kratzer misunderstood the object of Dr. Head's paper. I did not understand Dr. Head to say every gentleman advanced his method over all others, but others said they were correct without proving it; for instance, the advocate of gutta-percha says I am always correct, while he cannot always be. Another man says I am always successful with gold, and endeavors to give the impression to the society that he is.

It would seem that the object of Dr. Head's paper is to assist in putting a check on each one rising upon floors of associations and reading papers that assert results, without bringing forth statistical proof of the positions they take. I think if those who have been our leaders would give us statistical proof of their theories, young men and others would be better able to form a definite method of practice. As it is now, there is a great deal of matter given to the profession that is merely supposition, and we do not know who is correct. We have to accept the fact that the gentleman who asserts it believes it. Let us give them statistics or ocular demonstration, not having to prove that our theory is correct, but, if it is wrong, that it may be shown to be an error.

*Dr. Joseph Head*, Philadelphia.—One explanation more. When a man makes an experiment everybody knows that he is liable to error, and we know that when he makes a mistake he has not performed that experiment correctly, and therefore people, when they err by his experiments, may claim that his theory is unstable. We shall have to trace that error to its commencement by getting other dentists to make the same experiments, thus following out his operations. They will make errors themselves, but it will probably be in other parts of the experiment, and so, by having many men perform the same experiment, each endeavoring to arrive at the truth, the results in total will clearly demonstrate the correctness or instability of the theory in question.

*Dr. Joseph R. C. Ward*, Philadelphia.—I think Dr. Head's paper very forcibly presents to us, especially those who attend society meetings, what we frequently hear; and that is, members rising and making assertions that their mode of practice is an unqualified success. No matter what that mode may be, they still make their assertions, and I take it that Dr. Head's paper is not to uphold any particular method, but to condemn the practice of members who assert that what they do is always a success.

Now, it is not always the presentation of statistics that proves a method is successful. Two years ago a paper was read in which statistics were given in proof of the assertions, and, in addition, they brought specimens before the society, and yet nearly every member present condemned that practice. I take it that what one practitioner may do successfully another may not do. In the filling of root-canals some practitioners are more successful with gold, others with cotton, some with oxychloride, and others again with gutta-percha. Those who are successful with gold may not be successful with other materials. I think due allowance ought to be made for the practice that gives us success. If it is only one method, and we are successful with it, I think it should not be overlooked.

*Dr. C. V. Kratzer.*—Do not misunderstand me. I think it is wrong for any man to say he is successful in all cases, and I endorse that part of the paper which says "idle assertions should not be made." I merely want to refer to the impracticability of giving such proof at all times.

*Dr. Joseph Head*, Philadelphia.—I meant to say that whenever a person gives his testimony it should be as an opinion; but when a person states a fact, he should be compelled to prove it, either by experiment or by statistics. Let him give statistics to go on record. Other gentlemen will give similar evidence, and if their statistics agree, the conclusion will be that the statement is correct.

Subject passed.

DISCUSSION ON DR. KIRK'S PAPER ON "THE BONWILL METHOD OF PACKING GOLD FOIL."

*Dr. W. E. Magill*, Erie.—I would like to ask if, in packing gold over frail walls, you find an advantage in this method. As it first strikes me, it seems that in the power being applied from the centre to the surface the blow strikes at its weakest point. It should be from the outside to the centre instead of otherwise, but you have probably tested it, and do you find it the safer way of packing against frail walls?

*Dr. Edward C. Kirk*, Philadelphia.—In filling any cavity the gold should be in as close contact as possible. As I understand filling a cavity, whether the walls are frail or not, the gold should be packed against those walls. I do not think it is possible to get a perfect joint except in this way. In the use of the mallet in conjunction with the convex points, the instrument may at times come in contact with the enamel walls, but the rounded surface will follow over, and you will burnish or planish the gold into place in a uniform manner, and pack it up against the cavity-walls instead of chipping down on it.

*Dr. W. E. Magill*, Erie.—The success of this would depend very much on the nicety of manipulation, so that you do not bring undue pressure upon the edge. We always supposed that the success was in a vertical blow passing around the margin. While we condensed vertically we secured with the least amount of pressure against the walls of the cavity a tight joint. In packing by central force and pressing outward we feel that we should be taking additional risk, and now, as I say, I presume your idea is that as you manipulate outward with this sliding movement you must be very steady of hand and clear of eye, and so adjust your force to the weakness of that wall that you shall not break it?

*Dr. Edward C. Kirk*, Philadelphia.—To illustrate better the point I am bringing out, we will say the surface of the gold is extending out the left side of the cavity-wall. The operation will be the same as if I take my thumb and with a piece of tape of gold simply wipe it in over to one side, and then back again. I shall be satisfied if the gentlemen will be sufficiently interested in what I have said here to try for themselves the method I have shown,—using the rubbing-in movement with the Bonwill mallet. I had the Bonwill mallet six months in my office before this inspiration came to me. After thinking I had made a discovery I went around to Dr. Bonwill's office and told him about it. He laughed, and put in my hands a dozen points, proving that he had used it himself before. These points had not been made for sale, for the reason, he said, that the profession was so wedded to the dotting action that they would not change. It condenses the foil much better than any method I have seen. It is a percussive force.

*Dr. C. S. Beck*, Wilkesbarre.—I have no experience whatever with the mechanical mallet, but I claim to have a great deal of experience with the electric mallet. I have used it ever since it came out. I think Dr. Webb used the same method that Dr. Kirk mentions as originated by Dr. Bonwill. Dr. Webb always ran his instrument across the filling, but perhaps not to the extent that Dr. Kirk describes it. It looked like the dotting of a pin, and he always preferred a flat instrument and not a convex one. I can readily understand Dr. Kirk,—how he creeps along with the convex instrument; as he advances, the convexity of the instrument catches the gold and keeps it down. I have not tried that method, but I will do so. The next time I go to Philadelphia I shall take the liberty of calling on Dr. Kirk to see the instrument work. If it were flat it would sort of turn the gold up, but instead of this, being convex, it turns it down and against the walls. I think a great many failures are due to the manner in which we strike the blow. With the electric mallet you can get just as delicate a blow as you want. You do not want a sledge-hammer. If too much, instead of condensing, you chip it. I want to thank Dr. Kirk very much for the idea of convexity in the points.

*Dr. Edward C. Kirk*, Philadelphia.—I want to say here regarding the paper on this subject, published in the *Cosmos* and read before the First District Society,—"American System against Herbst,"—that no man would take sides against it. I have had so much comfort in it that I said I would come before the society and give them the benefits of my experience.

*Dr. W. B. Miller*, Altoona.—I cannot conceive how any one can manipulate gold with anything better than the electric mallet. I have seen the Bonwill mallet in use. My experience is that with the Bonwill there is too much vibration. With the electric you have perfect control over the instrument, and no weight. I do not altogether agree with Dr. Kirk as to the method of Dr. Webb in packing gold. It is not as a pin, but simply is a "dotting over." There is great room for improvement in the shapes of instruments for the mallet. Only two or three are of any value to me. I would like to ask Dr. Kirk if he cannot manipulate gold as well with the electric as with the Bonwill mechanical.

*Dr. Edward C. Kirk*, Philadelphia.—The instrument I have now I did not have when I tried the electric. I think I can work much faster with the Bonwill mechanical mallet. In regard to Dr. Webb's method, I think Dr. Miller is correct. I think he used an approximation of this, and Dr. Bonwill was then working it out. Webb used this dotting action of the pin with the mallet, and as soon as he got the idea of wiping he commenced to use it, but I do not think he arrived at the same perfection.

*Dr. H. E. Roberts*, Philadelphia.—Does not Dr. Bonwill's method resemble Dr. Fillebrown's way of pushing gold into position? He does not get a blow at all, but with the same rounded instruments packs the gold into position.

*Dr. J. A. Libbey.*—Last year, in Chicago, Dr. Stevens showed me his filling instruments, which were of very peculiar construction. They were convex-shaped instruments. He took these instruments and had them serrated down the side also. In the method of manipulation touched upon here, he said it was a great improvement. Now, Mr. President, Dr. Miller says there is no instrument in his hands that he can use with the fine, delicate manipulation he can the electric mallet. In my hands there is nothing I can use with the same delicate touch as the hand-mallet. I have tried to use the electric mallet, and with me it is a failure. I have tried nearly every other method except the mechanical. This all illustrates the fact that whatever instrument or method we thoroughly educate ourselves in, this will be the most successful in our hands, each variety tending to the same good result,—that of the successful manipulation of gold. A word further in this connection. I believe the doctor said he used semi-cohesive gold in starting. My experience is that I cannot make a successful operation with either cohesive or semi-cohesive gold on approximal surfaces. I believe I have had some satisfactory results in that way, but I have had a great deal more success with soft gold at the cervical border than with cohesive. I have concluded that I cannot fill approximal cases as well with semi-cohesive as with soft.

*Dr. C. S. Beck*, Wilkesbarre.—I think that we as dentists get hold of that sliding backward-and-forward movement by thinking of Dr. Herbst. I got a set of points, and went home and tried several fillings with his method. Some I was satisfied with. The other day I saw two that I had inserted by the Herbst method, and was very well satisfied with them. I thought, however, that the rotary motion took up more time than if I used the mallet, and did not do the work as well as the latter instrument. Dr. Webb always went from the centre to the periphery, and was always careful to take the gold over the edge of the cavity. I am much pleased with this method, and I think it is good.

*Dr. W. B. Miller*, Altoona.—I think it would be well to suggest to the dental manufacturers to make points after the manner you mention.

*Dr. Edward C. Kirk*, Philadelphia.—The Whites make a set of points such as I use. They are half Dr. Bonwill's and half my own. The reason I should not use them in the same way with the electric as with the mechanical mallet is that the blows are different. I want it distinctly understood that I lay no claims to originality. It is simply a method based upon Bonwill's. He devised it, and is entitled to full credit.

Subject passed.

(To be continued.)

